# Proliferation, steroid receptors and clinical/pathological response in breast cancer treated with letrozole

**DOI:** 10.1038/sj.bjc.6603001

**Published:** 2006-03-14

**Authors:** W R Miller, S White, J M Dixon, J Murray, L Renshaw, T J Anderson

**Affiliations:** 1 The Edinburgh Breast Unit, Western General Hospital, Edinburgh EH4 2XU, UK

**Keywords:** breast cancer, neoadjuvant therapy, aromatase inhibitor, proliferation, steroid receptors, clinical response

## Abstract

Sixty-three postmenopausal women with large primary breast cancers were treated with neoadjuvant letrozole (2.5 mg daily) for 3 months. Tumour samples were taken at diagnosis and after 10–14 days and 3 months treatment. Immunohistochemical staining for Ki67, oestrogen receptor (ER) and progesterone receptor (PgR) was performed and related to clinical (ClinR) and pathological responses (PathR) after 3 months treatment. ClinR was observed in 48 of 63 cases (76.2%) and PathR in 47 of 62 (75.8%). Pretreatment Ki67 scores were similar in responders (R) and non-responders (NR). Highly significant Ki67 decreases occurred in all tumour subgroups at 10–14 days (*P*<0.005). A significant difference in Ki67 scores at 10–14 days (*P*<0.007) was found between PathR and PathNR but not between ClinR and ClinNR. At 3 months, decreases from pretreatment Ki67 scores were highly significant in all tumour subgroups irrespective of response status. However, whereas Ki67 scores were significantly different between pathological R and NR (*P*=0.009), the corresponding comparison of ClinR status was not. Significant decreases between 10–14 days and 3 months were found only in ClinR and PathR (*P*=0.02 and 0.045, respectively). Treatment significantly reduced PgR expression at 14 days and 3 months (both *P*<0.0001), but the level of changes was not different between response status groups. In summary, letrozole produces rapid and profound decreases in expression of Ki67 and PgR but changes do not always correlate with clinical and pathological responses.

Letrozole is a third-generation aromatase inhibitor, which in clinical trials has been shown to be highly effective in postmenopausal women with oestrogen receptor (ER)-positive breast cancer (Eiermann *et al*, 2001; Mouridsen *et al*, 2001; Goss *et al*, 2003; Rose *et al*, 2003). Previous studies using neoadjuvant therapy have shown that letrozole may produce profound changes in tumour pathology and immunohistochemical markers (Dixon *et al*, 2001; Ellis *et al*, 2001, 2003; Miller *et al*, 2003; Anderson *et al*, 2004). Furthermore, it is clear that the clinical effects of neoadjuvant treatment with third-generation aromatase inhibitors in postmenopausal women are not dissimilar to those seen with neoadjuvant chemotherapy (Dixon *et al*, 2001) and are achieved with less morbidity. Comparative studies have also shown that the effects of third-generation aromatase inhibitors are more consistent and greater than tamoxifen on proliferation (as measured by Ki67) and markers of oestrogen action (progesterone receptors (PgR) and trefoil factor 1) (Ellis *et al*, 2003; Miller *et al*, 2003; Anderson *et al*, 2004). It is therefore interesting that in the neoadjuvant setting, letrozole yields significantly superior clinical results than tamoxifen and also appears to be more effective in particular subgroups such as tumours with low ER levels and overexpression of HER-2 (Ellis *et al*, 2001). However, the timescale of these effects and their relationship to clinical and pathological response as assessed at the end of treatment has yet to be fully defined. The aim of the present study was therefore to assess the effects of letrozole on the proliferation marker Ki67 and receptors for oestrogen and progesterone by immunohistochemical assessment in serial biopsies from primary breast cancers taken before, at 10–14 days and at 3 months into treatment.

## MATERIALS AND METHODS

### Patients

A total of 63 postmenopausal women presented to the Edinburgh Breast Unit with large (>3 cm) primary breast cancer, which were ER-rich (Allred score 5–8). (However, review of the cases in the research laboratory showed that all patients recruited to the study had ER scores of 7 or 8.) All patients apart from 12 were technically operable. The primary clinical objective was to downstage tumours such that those who were inoperable became amenable for surgery and those who would have required mastectomy could become candidates for breast conservation. This series represents consecutive patients recruited but excluding cases in which the tumour was shown to be multifocal or of special histological type (e.g. mucinous, tubular/cribriform and lobular). All patients gave informed consent to be included in the study, which had been approved by the local ethics committee (2001/W/BU/09 and 2001/W/BU/10).

### Treatment

All patients received letrozole (2.5 mg, daily) for 3 months. Serial measurements of the primary tumours were taken before, at 6 weeks and at 3 months by calliper and ultrasound as described previously (Forouhi *et al*, 1994; Dixon, 2001). The tumour was also imaged mammographically before and at 3 months. Core biopsies were taken at the start, after 10–14 days and at 3 months of treatment as described previously (Iqbal *et al*, 2002). All patients, apart from eight patients (who electively continued on treatment), received definitive surgery at 3 months.

### Response assessment

Tumour volumes were determined from ulstrasound measurements as described by Forouhi *et al* (1994). Reduction in volume over a 3 month period >50% was regarded as clinical response; this includes both complete and partial responders.

Pathological response was determined by comparing biopsies taken before and after 3 months of treatment. Only marked reduction in cellularity and/or a clear increase in fibrosis were used as evidence of pathological response. Although these changes are essentially subjective, they were confirmed by two observers working independently. The criteria may underestimate actual morphological changes occurring to a lesser degree, but these were excluded because of the difficulty in comparing histological appearances on limited tissue such as in core biopsies with those in the more substantial material obtained at tumour excision. No case was classified as a complete PathR, residual evidence of malignant cells being evident.

### Immunohistochemistry

Immunohistochemistry staining with antibody to MIB1 (Ki-67) antigen (Europath Ltd, Cornwall, UK) diluted × 50 was used as a measure of tumour cell proliferation. Reactivity was detected by an ABC–peroxidase–antiperoxidase (PAP) method, and scored according to the method described by Going (1994). A change of >40% between different paired biopsies was taken as being meaningful (Ellis *et al*, 1998; Iqbal *et al*, 2002) and a value of <1% was regarded as indicating a lack of proliferation.

Reactivity for ER or PgR was performed by the PAP method, after microwave antigen retrieval, using ER*α* antibody clone 6f11/2 (Novocastra Ltd, Newcastle, UK) and PgR antibody clone PgR636 (DAKO Labs, Ely, UK) using the DAKO EnVision system according to the manufacturer's instruction. Results were scored on a scale of 0–3 for staining intensity (with each successive score denoting increasing intensity), and on a score of 0–5 for increasing proportion of positive cancer nuclei (0=none, 1=<1%, 2=1–10%, 3=11–33%, 4=34–66%, 5=>66%). The values were then summed into a category score within a range of 0–8 (Allred *et al*, 1998).

### Statistics

Non-parametric comparisons using either Wilcoxon rank or Spearman paired testing was employed and, where appropriate, 3 × 2 *χ*^2^ testing.

## RESULTS

### Clinical and pathological response

Of the 63 patients, 48 (76.2%) were classified as clinical responders (ClinR). With regard to pathological response, one case was not assessable because of insufficient material after biopsy at 3 months. Of the remaining 62 cases, 47 (75.8%) had clear evidence of pathological response. Although response rates were similar, there was not exact concordance between clinical and pathological outcomes. Thus, 42 of 48 ClinR were also pathological responders (PathR) (six were pathological non-responders (PathNR)) and nine of 15 clinical non-responders (ClinNR) were also PathNR. Of the remaining six cases, five were PathR and one case was pathologically not assessable.

### Proliferation (Ki67)

The tumour Ki67 scores for pretreatment, 10–14 days and 3 months samples, subdivided according to clinical and pathological response status, are shown in Table 1.

No significant differences were apparent for pretreatment group scores between responders (R) and non-responders (NR) whether assessed clinically or pathologically. However, at 10–14 days, whereas there was no difference between ClinR and ClinNR, the PathNR were significantly higher than PathR (*P*=0.024). Similarly at 3 months, although there was no significant difference between ClinR and ClinNR, values in PathNR were significantly higher than in PathR (*P*=0.009).

With regard to group changes in Ki67 with treatment, the scores at 10–14 days and 3 months were highly significantly decreased, as compared with those in paired pretreatment biopsies. These changes were irrespective of clinical or pathological response status. In terms of changes occurring between 10–14 days and 3 months, a significant decrease was seen in ClinR (*P*=0.02) and PathR (*P*=0.045). However, values were not significantly different between 10–14 days and 3 months in ClinNR and PathNR.

Changes in Ki67 with treatment in individual tumours are summarised in Table 2. In terms of >40% change criteria for ClinR, 52 of 63 cases (82.5%) showed a decrease at 10–14 days, and 53 of 63 tumours (84%) showed a reduction at 3 months. These reductions were seen in both ClinR and ClinNR groups, and there was no significant difference between them. However, for pathological assessments, there was a significant difference between PathR and PathNR at 10–14 days (*P*=0.034) but not at 3 months. Treatment effects were also assessed on the basis of reducing Ki67 scores to <1%. It can be seen that whereas 11 cases (17.5%) were reduced to <1% by 10–14 days, these numbers markedly increased to 27 (42.9%) by 3 months. Whereas the reduction to <1% was seen in both responding and non-responding tumours, the increase in numbers between 10–14 days and 3 months is predominantly associated with responding tumours.

**Figure 1:**
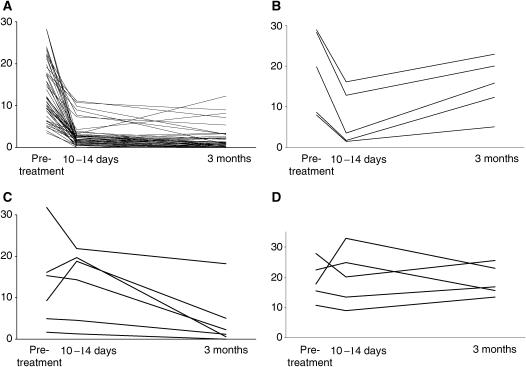
Tumour Ki67 scores before and after 10–14 days and 3 months treatment with letrozole. (**A**) Cases that show decreases (>40%) at both 10–14 days and 3 months. (**B**) Cases that show decreases (>40%) at 10–14 days but not at 3 months. (**C**) Cases that show no change at 10–14 days but a decrease (>40%) at 3 months. (**D**) Cases that show no decrease at either 10–14 days or 3 months.

**Figure 2:**
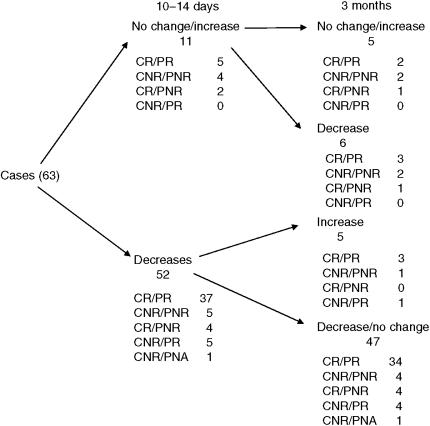
Flow diagram indicating number of cases grouped according to Ki67 changes at 10–14 days and 3 months and further subdivided according to final clinical/pathological response.

Different patterns of changes in Ki67 over the treatment period could be detected. These are shown in Figure 1. The largest cohort of tumours (47) showed substantial decreases at 10–14 days, which were maintained or fell further at 3 months (in these tumours, changes in Ki67 at 10–14 days were predictive of those at 3 months). However, there was a small cohort of five patients in which a decrease at 10–14 days was followed by a substantial rise in score at 3 months (in these tumours, changes in Ki67 at 10–14 days did not therefore concur with those at 3 months). Of the 11 that did not decrease at 10–14 days, six decreased at 3 months (Ki67 changes therefore did not concur at 10–14 days and 3 months) and five were not reduced at 3 months (lack of change in Ki67 at 10–14 days was predictive of no change at 3 months). These patterns of Ki67 did not correlate with ClinR and PathR phenotype (Figure 2).

### Progesterone receptors

Of the 63 cases, 57 (90%) were PgR+ve; of the six negatives, five were ClinR and four were PathR. The changes with treatment of PgR staining subdivided according to ClinR and PathR status are summarised in Table 3.

Treatment reduced PgR scores such that, at 10–14 days, values were significantly lower than those in the paired pretreatment biopsy (*P*<0.0001). This decrease was found in all tumour subgroups irrespective of clinical or pathological assessment status. Similar highly statistically significant decreases were also seen when pretreatment values were compared with corresponding pairs at 3 months (*P*<0.0001). Of note is the high proportion of positive tumours that decreased to 0 by 10–14 days (45.6%). This percentage rose to 66.7% at 3 months. A comparison of 10- to 14-day biopsies with those at 3 months showed significant decreases with extended treatment with the groups of ClinR and PathR, whereas no significant difference was detected in the NR group (but this represents a small number of pairs). In terms of comparisons between tumours R or NR assessed either clinically or pathologically, no significant differences were detected either at pretreatment, 10–14 days or 3 months in absolute values (data not shown).

### Oestrogen receptors

There was no significant difference in ER score pretreatment, after 10–14 days and after 3 months. Neither was there a significant change with treatment with all biopsies scoring 7 or 8 throughout (data not shown).

## DISCUSSION

The observation that neoadjuvant treatment with letrozole is associated with a marked reduction in the immunohistochemical expression of Ki67 and PgR confirms our previous findings (Miller *et al*, 2003; Anderson *et al*, 2004) and those of others (Ellis *et al*, 2001, 2003). However, the present study extends our previous work by demonstrating that such effects are evident as early as 10–14 days into treatment in over 80% of cases. Similar results have recently been presented for anastrozole (Dowsett *et al*, 2005a, 2005b). The same group have also presented results from a randomised neoadjuvant trial comparing the aromatase inhibitor vorozole with tamoxifen. Ki67 levels fell within 2 weeks of treatment and remained suppressed at surgery 3 months later (Harper-Wynne *et al*, 2002). These effects are therefore apparent before evidence of morphological changes in tumour pathology and clinical evidence of changes in tumour volume. It was of interest in the present paper to determine whether changes in proliferation and PgR expression related to and/or predicted for subsequent ClinR and PathR.

In terms of assessing proliferation status with Ki-67 scores, we have analysed results in three ways: (i) comparison of tumour scores at individual time points grouped according to response status at 3 months, (ii) classifying a >40% change in Ki-67 between different time points as evidence of a meaningful change in proliferation and (iii) comparing the number of cases in which proliferation is reduced to <1%, a value that we have regarded as a state of virtual non-proliferation. By using these multiple analyses, we hoped to derive impressions not only of group trends but effects and degree in individual cases.

Group comparisons of mean Ki-67 scores at individual study time points revealed interesting differences according to whether response was assessed clinically or pathologically. Thus, the only detectable significant differences were in groups subdivided by pathological assessment in which higher mean scores were found in non-responding tumours at both 10–14 days and 3 months. Interestingly, the same general pattern was evident when categorising individual cases according to >40% reduction (the only significant difference was seen between PathR and PathNR at 10–14 days). The restriction of significant effects to pathological assessment probably reflects the closer association between two histological assessments rather than that between histology and tumour size. It is also worth noting that the tumour morphology after treatment will be determined by factors in addition to proliferation, such as cell loss. In this respect, although Ki67 is a primary marker of proliferation, it can also be a secondary reflection of cell death (Archer *et al*, 2003).

A reduction of >40% in Ki-67 was apparent in most cases at 10–14 days, and extended treatment to 3 months was associated with only minor changes in the proportion of tumours that displayed a >40% decrease in proliferation. This is in contrast to the results based upon the more profound criteria of a decrease to an absolute value of <1%. These results show that, remarkably, even after 10–14 days of treatment, 17% of tumours have reached this state of virtual non-proliferation, and this was irrespective of whether tumours subsequently displayed evidence of clinical or pathological response. However, the proportion of cases falling to <1% proliferation increases further to 43% by 3 months. Interestingly, this incremental effect with time appears restricted to those tumours that had either a pathological or clinical response status at 3 months. It is clear that letrozole is capable of producing increased suppression of proliferation when used over an extended period.

The overall perspective therefore is that letrozole is capable of producing a rapid reduction in tumour proliferation that is seen in most tumours irrespective of subsequent clinical and pathological response, but that incremental effects on proliferation (as monitored by scores of <1%) are additionally seen in the period between 10–14 days and 3 months, and these are largely restricted to PathR or ClinR.

Whereas changes in Ki-67 levels have been revealed by group comparisons, the strength of neoadjuvant studies is that it is possible to examine differences in individual cases and classify tumours according to sequential changes in proliferation. Thus, consistent with the general trends discussed above, most tumours displayed a substantial decrease in proliferation >40% by 10–14 days and this was sustained at 3 months. However, it was also possible to identify (i) a small cohort that initially had decreased proliferation at 10–14 days but which largely disappeared by 3 months, (ii) tumours that failed to demonstrate a decrease in proliferation at 10–14 days but had a delayed decrease apparent at 3 months and (iii) cases that failed to display a decrease in proliferation at both 10–14 days and 3 months. It is therefore important not only to discuss relationships between clinical/pathological response and proliferation at individual time points, but also to take into account the patterns of change in response to treatment.

Statistically significant differences were detected between PathR and PathNR in (i) group levels of Ki67 at 10–14 days and (ii) the proportion of cases decreasing in Ki67 >40% between pretreatment and 10–14 days. However, there was a large overlap in values at 10–14 days between PathR and PathNR, and individual tumours could display an increase, no change or a decrease in Ki67 irrespective of being PathR or PathNR. Consequently, measurements of Ki67 in individual cases do not accurately predict for subsequent pathological (or clinical) response. A similar lack of prediction between Ki67 changes and clinical response to anastrozole has been observed in the recently reported IMPACT neoadjuvant trial (Dowsett *et al*, 2005a) (although the short-term changes in proliferation did parallel recurrence-free survival between the three treatment groups, anastrozole, tamoxifen and Arimidex combined with tamoxifen) (Dowsett *et al*, 2005b). As a consequence, consideration therefore needs to be given as to why clear decreases in cellular proliferation at 10–14 days do not translate into pathological response and why conversely responding cases can show no change or even an increase in Ki67 with treatment.

It is possible that lack of correlation in some cases relates in part to imprecise measurements of proliferation or misclassification of response. In terms of immunohistochemical assessment of Ki67, we have already published data on reproducibility in breast cancer biopsies (Iqbal *et al*, 2002). These showed that, because of inherent heterogeneity, marked variation in Ki67 score may be observed in the same tumour without intervening treatment. However, this is restricted to occasional tumours, and the number of cases in the present study with discordance between proliferation changes and clinical/pathological response is greater than would have been expected. Furthermore, in order to reduce spurious results, we have used three different criteria for assessing changes in Ki67. In terms of the impact of assessment of clinical response, potential sources of inconsistencies have been considered elsewhere and vary according to the technology employed (Forouhi *et al*, 1994). In the present studies, clinical responses were based primarily upon ultrasound measurements, but they were substantiated by parallel calliper and mammographic measurements in all cases. For ease of presentation, clinical response was dichotomised and it is possible that the use of continuous variables might have been more informative. However, preliminary analyses using continuous variables did not reveal better relationships between proliferation and response (data not shown). There are also limitations to the assessment of pathological response in that the pretreatment assessments (and some of the post-treatment) were performed on core biopsies, which are not guaranteed to be representative of the total tumour mass. It is also possible that assessment of ClinR/PathR at the single time point of 3 months is associated with chronological inaccuracy in that certain tumours classified as NR may go on to respond with extended treatment (Dixon *et al*, 2005). There is no doubt that response is not complete by 3 months and treatment up to 12 months may be associated with (a) further tumour shrinkage and (b) an increased incidence of complete ClinR.

Although methodological imprecision might be influential in some cases, it is unlikely that these totally account for the lack of association between proliferation and response. Other reasons need to be considered including the possibility that reduction in proliferation alone may not produce tumour shrinkage and cell death or apoptosis may be equally influential. Although we have not measured apoptosis in tumour samples from the present study (because assessment in core biopsies was not sufficiently reproducible), we did not find apoptosis to be predictive of response in other tumour samples from patients offered neoadjuvant endocrine therapy (Anderson *et al*, 2002). However, this may be because differences are small and transient; primary effects on apoptosis may also be masked by those occurring secondarily (e.g. as a result of decreased proliferation).

Another cause for a reduction in proliferation at 10–14 days not translating into subsequent tumour response could be that the effect is transient and not maintained over a sufficiently extended period to produce tumour shrinkage. However, in the present study, four of the five non-responding tumours with a reduction in Ki67 had a sustained decrease in proliferation to 3 months. A disconnect between changes in proliferation as observed at 10–14 days with subsequent clinical/pathological response could also be explained if the proliferation response was delayed. Interestingly, six of 11 tumours showed a delayed reduction in Ki67 scores; three of these were classified as PathR/ClinR.

It is worth noting that in 11 of 63 (17%) cases, change in proliferation at 10–14 days failed to predict that at 3 months. Furthermore, most importantly, change in proliferation at 10–14 days failed to predict clinical response in 18 out of 63 (29%) cases and pathological response in 14 out of 62 (23%) tumours. Change in proliferation at 10–14 days is therefore not an accurate surrogate of clinical/pathological response at 3 months.

The other marker that showed major changes with therapy was PgR. Thus, 78% of cases displayed a reduction of at least 1 category score by 10–14 days and was maintained to 3 months. Reduction occurred irrespective of subsequent clinical/pathological response. The extent of effect may be gauged by the percentage of cases reduced to negativity (40% at 10–14 days and 60% at 3 months); again the decrease to negativity was irrespective of clinical or pathological response. As PgR is a marker of signalling from the ER, these changes are clear evidence of the anti-oestrogenic effects of letrozole and contrast with those of tamoxifen (Anderson *et al*, 2002; Miller *et al*, 2003).

Although we have not formally presented the correlations between changes in PgR and proliferation, there was a positive relationship between them. However, there were instances of discordance whereby at both 10–14 days and 3 months, tumours displayed either a phenotype of reduced proliferation but stable PgR or unchanged proliferation and reduced PgR. As reduced PgR expression is a marker of oestrogen deprivation, it is unlikely that the lack of effect on Ki67/ClinR/PathR in these cases is because of lack of aromatase inhibition. Hence, changes in PgR with treatment, although marked and occurring early, are not predictive of clinical or pathological response. It was also of interest to examine changes in PgR score in tumours that initially displayed a decrease in Ki67 at 10–14 days but an increase at 3 months. This phenotype could reflect adaptive changes leading to an oestrogenic stimulation or a state of hypersensitivity to oestrogen (Santen *et al*, 2004); however, changes in PgR in this cohort of five tumours with this phenotype revealed three cases that decreased between 10–14 days and 3 months and two cases that were negative at all time points, providing no evidence for adaptive changes or hypersensitivity to a reduced oestrogenic environment.

In conclusion, the present study has provided evidence that neoadjuvant letrozole produces marked effects on levels of Ki67 and PgR within 10–14 days. Although early changes in proliferation are less likely to occur in tumours that show no pathological response at 3 months, the effects can be seen irrespective of clinical and/or pathological response in individual cases. Ki67 scores and PgR expression are therefore of limited value as predictors of response. They do however reflect the potent anti-oestrogenic and anti-proliferative properties of the third-generation aromatase inhibitors and it has been suggested that such changes may relate to long-term outcome (Ellis *et al*, 2003; Dowsett *et al*, 2005b).

## Figures and Tables

**Table 1:** Ki67 scores in tumour taken before and after 10–14 days and 3 months of treatment, subdivided according to response status

	**Ki67 scores (mean±s.e.m.)**		
	**Pretreatment**	**10–14 days**	**3 months**
Clinical responders (48)	14.17±1.10	5.11±0.98^+^	4.13±0.96^+,♦^
Clinical non-responders (15)	15.29±2.06	6.72±2.04^+^	5.85±1.91^*^^,^°
	*P*=0.70	*P*=0.77	*P*=0.13
Pathological responders (47)	14.03±1.09	4.02±085^+^	3.47±0.90^+,♦♦^
Pathological non-responders (15)	15.97±2.22	9.35±2.22^**^	8.11±2.01^***^^,^°
	*P*=0.52	*P*=0.024	*P*=0.009

Compared with pretreatment tumour: ^+^*P*<0.0001; ^*^P=0.003; ^**^P=0.007; ^***^P=0.009.

Compared with tumour taken after 10–14 days of treatment: ^♦^*P*=0.02; ^♦♦^*P*=0.045: °*P*=NS.

**Table 2:** Changes in tumour Ki67 scores with treatment

	**No. of patients**							
	**Changes at 10–14 days**				**Changes at 3 months**			
	**Increase**	**No change**	**Decrease**		**Increase**	**No change**	**Decrease**	
			**by 40%**	**to <1%**			**by 40%**	**<1%**
Clinical responders (48)	1	6	41	(8)	0	7	41	(23)
Clinical non-responders (15)	1	3	11	(3)	1	2	12	(4)
Total	2	9	52	(11)	1	9	53	(27)
			*P*=0.49				*P*=0.20	
								
Pathology responders (47)	1	4	42	(9)	0	6	41	(23)
Pathology non-responders (15)	1	5	9	(2)	1	3	11	(3)
Total	2	9	51	(11)	1	9	52	(26)
			*P*=0.034				*P*=0.15	

Statistical comparison of increase (>40%), decrease (>40%) or no change (<40%) compared with pretreatment values by 3 × 2 chi-square testing.

**Table 3:** Changes in tumour PgR score with treatment

	**No. of patients**							
	**Changes at 10–14 days**				**Changes at 3 months**			
	**Increase**	**No change**	**Decrease**	**(Decrease to 0)**	**Increase**	**No change**	**Decrease**	**(Decrease to 0)**
Clinical responders (48)	1	11	36	(18)	0	9	39	(29)
Clinical non-responders (15)	0	2	13	(8)	0	2	13	(9)
								
Pathology responders (47)	1	11	35	(16)	0	9	38	(26)
Pathology non-responders (15)	0	2	13	(9)	0	2	13	(11)

PgR=progesterone receptor.
